# A portable and affordable aligner for the assembly of microfluidic devices

**DOI:** 10.1016/j.ohx.2022.e00348

**Published:** 2022-08-27

**Authors:** Victoria Guglielmotti, Nicolás Andrés Saffioti, Ana Laura Tohmé, Martín Gambarotta, Gastón Corthey, Diego Pallarola

**Affiliations:** Instituto de Nanosistemas, Universidad Nacional de General San Martín, San Martín, Provincia de Buenos Aires, Argentina

**Keywords:** Microfabrication, Mask aligner, Sensors, Electrochemistry, Soft Lithography

## Abstract

The incorporation of sophisticated capabilities within microfluidic devices often requires the assembly of different layers in a correct arrangement. For example, when it is desired to include electrodes inside microfluidic channels or to create 3D microfluidic structures. However, the alignment between different substrates at the microscale requires expensive equipment not available for all research groups. In this work, we present an affordable, compact and portable aligner for assembling multilayered composite microfluidic chips. The instrument is composed of aluminum machined pieces combined with precision stages and includes a digital microscope with a LED illumination system for monitoring the alignment process. An interchangeable holder was created for substrate fixing, allowing the bonding of PDMS with other materials. Microscopic visualization is achieved through any device with internet access, avoiding the need of a computer attached to the aligner. To test the performance of the aligner, the center of an indium tin oxide microelectrode on a glass substrate was aligned with the center of a microchannel in a PDMS chip. The accuracy and precision of the instrument are suited for many microfluidic applications. The small and inexpensive design of the aligner makes it a cost-effective option for small groups working in microfluidics.

## Graphical abstract

**Figure:**
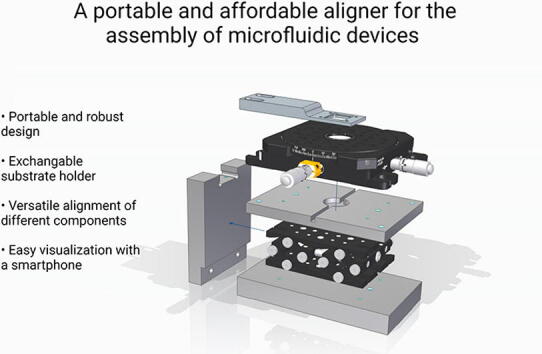

Specifications table.

**Table:** 

Hardware name	Portable and affordable aligner for the assembly of microfluidic devices
Subject area	• Engineering and materials science • Chemistry and biochemistry • Biological sciences
Hardware type	• Mechanical engineering and materials science
Closest commercial analog	*FINEPLACER pico ma (Multi-Purpose Bonder) FINETECH GmbH*
Open source license	*GNU General Public License (GPL) version 3.*
Cost of hardware	*$ 2065*
Source file repository	http://dx.doi.org/10.17632/jpxw5dph27.1

## Hardware in context

In recent years, the use of microfluidic devices has grown considerably, becoming useful platforms for the study of biological processes [1]. Soft lithography is one of the most popular methods for fabricating microfluidic devices and consists in generating polydimethylsiloxane (PDMS) replicas of a microchannel array from a mold of photoresist in silicon wafers [2]. The PDMS replicas are adhered to silicon, glass or other PDMS substrate by an O_2_ plasma treatment that forms silanol (SiOH) groups on the surface. The activated PDMS is immediately placed in contact with the substrate to form Si-O-Si bonds at the interface. This creates a strong bonding between the PDMS and the substrate, ensuring a tight seal of the microfluidic channels [3].

There are many cases in which two or more layers must be aligned for the creation of a microfluidic device, for example, when microfluidics are used in biosensors. A biosensor is a device capable of detecting and measuring biological parameters using a biological recognition element and a transducer [4]. To incorporate these elements inside the microfluidic channels, composite devices are fabricated combining PDMS chips with electrodes on glass or silicon substrates [5]. Additionally, the creation of 3D microfluidic devices usually requires the correct assembly of several layers of PDMS or other materials [6]. When microfluidic devices are combined with micropatterning, the correct alignment between the micropatterned substrate with the PDMS chip is critical for the success of the experiments [7].

The alignment of different layers in microfluidic devices is not a trivial procedure and requires specific equipment. Mask aligners can perform this task, however they are expensive, restricted to cleanroom facilities and not designed for the alignment of PDMS substrates [8]. Commercial systems available for the assembly of multilayered microfluidic devices are available. For example, Mutech Microsystems (Argentina) fabricates an aligner system called Mutech microaligner designed for the assembly of multilayer devices that can be used for microfluidic applications. Finetech GmbH (Germany) commercialize the table-top flip chip bonder FINPLACER®, which is designed for the alignment and bonding of different components in microfabrication processes. Wenhao (China) offers an instrument designed for the assembly of microfluidic chips by bonding a PDMS layer with other substrate like glass, poly(methyl methacrylate) (PMMA) or PDMS. Although these aligners provide enough precision, they are costly and therefore not affordable to all research groups.

Custom made aligners have been proposed based on different approaches, including the use of high precision stages [9], capillary forces within the microfluidic chips [10], pluggable modules [11], magnets [12], and adapters to microscopy objectives [13]. Li et al. have designed a precise and low-cost aligner for the fabrication of multilayered PDMS devices [14]. In their design, the PDMS was attached to a glass plate on the upper position whereas, the rigid substrate is placed below on a moving Z-stage. Kipper et al. created an instrument capable of aligning PDMS with other materials since it incorporates a holder for one of the layers to be bonded. However, the design is not open source and incorporates a sophisticated semiautomatic method of aligning, making it not easily affordable [15]. Mou et al. have created an effective and low-cost hinge-based aligner, but it has the limitation that the two layers to be bonded must have a defined and precise size [16]. This hampers the use of their approach to PDMS chips created by typical soft lithography in which, the PDMS is cut manually. He et al. have created a specific design for the alignment of PDMS-PDMS chips with high precision, in which one of the PDMS chips is held in the upper position by reversible adhesion to a glass plate [17]. This is possible given the ability of non-plasma treated PDMS to reversibly bond to glass. However, this can be a limitation for the alignment of other type of materials. In addition, none of the mentioned works have provided the plans and instructions for the fabrication of the instruments.

Even though the cited works describe aligner designs that are well suited for many applications, we found them not convenient for the alignment of PDMS with transparent rigid substrates, like electrodes on glass. In these cases, including a holder for the rigid substrate allows to keep it motionless in the upper position, which has important advantages for many applications: (i) the microscope focus does not need to be adjusted in each alignment, as rigid substrates usually have the same thickness; (ii) transparent electrodes are generally thinner (thickness of 1 mm or less) than PDMS substrates allowing to work with conventional short working distance objectives; (iii) There is no need of a vacuum system to hold the rigid substrate in place. A comparison between our approach and the related ones found in the literature is presented in Supplementary Table 1.

Although there are several aligners proposed in the literature, a low-cost option for the fabrication of microfluidic devices combining rigid substrates with PDMS chips is currently lacking. To provide a solution for this need, we have created a portable aligner capable of producing PDMS-rigid substrates microfluidic devices with relatively good precision. The aligner includes a holding arm where a rigid substrate can be held in position during the alignment process. We have worked on a compact and transportable design that would be convenient for limited spaces. Our aligner was suitable for the fabrication of microfluidic sensors consisting of the assembly of a microelectrode fabricated on indium tin oxide (ITO) coated glass substrate [18] with a PDMS chip. The position of 50- and 100-μm-diameter ITO microelectrodes could be satisfactorily controlled inside a 250-μm-width microfluidic channel with adequate accuracy and precision.

## Hardware description

**Fig. 1:**
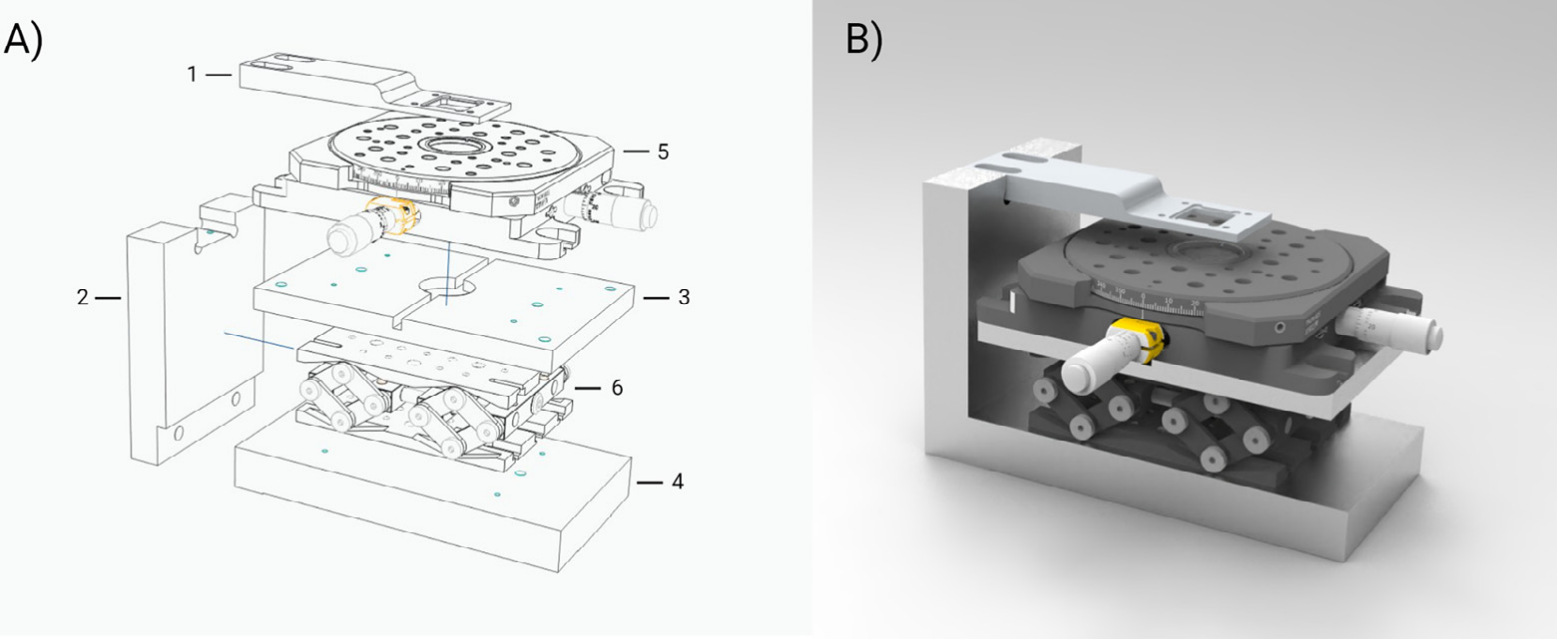
In panel A, the image shows the exploded view of the aligner with the aluminum machined pieces and stages: 1. Holding arm. 2. Arm support. 3. Stage adapter. 4. Base. 5. X/Y/θ-stage. 6. Z-stage. In panel B, the image shows a render of the assembled aligner (the digital microscope, the microscope holder and the Raspberry Pi are not included).

A critical aspect of the fabrication of a multilayer microfluidic device is the precise control of the position of the layers during the alignment. To hold PDMS layers, many aligners in the literature rely on the reversible adsorption of PDMS to glass surfaces. Thus, glass slides are incorporated in the alignment mechanism whose function is to hold the PDMS layers during the aligning process [14], [17], [15]. However, a different holding mechanism is required when aligning substrates of other materials. The aligner presented here incorporates a holding arm with a holder designed to support a rigid substrate in the upper position during alignment (Fig. 1). The rigid substrate is aligned with a PDMS layer that is placed on a glass slide on the X/Y/θ-stage. In our design, alignment is achieved by moving the PDMS layer, whereas the substrate in the holding arm stay motionless. The movement of the PDMS layer is performed by the X/Y/θ and Z-stages (Fig. 1). The use of a high precision X/Y/θ-stage allows for the adjustment of the PDMS layer position with µm precision. The aligner includes a low-cost USB-digital microscope with LED illumination with a resolution below 10 µm for monitoring the aligning process (Fig. S1). In combination, the X/Y/θ-stage and the microscope provide adequate accuracy for aligning ITO microelectrodes with PDMS microchannels as detailed in section 6.

**Fig. 2:**
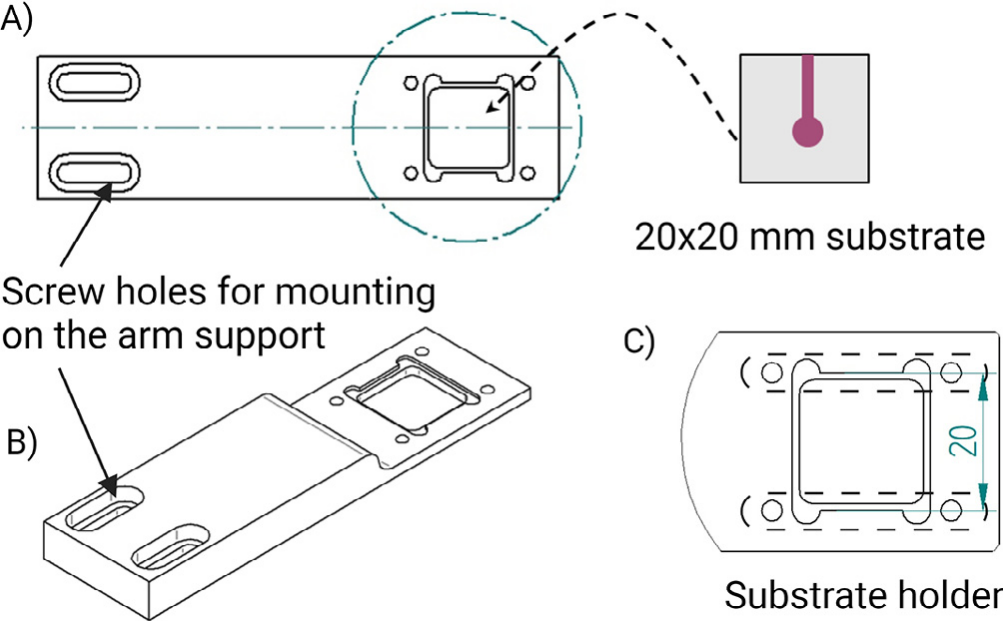
Holding arm for substrate holding. In panel A, the image shows a detailed drawing of the holding arm for 20x20 mm substrates (top view). Note that the substrate holder (indicated by a blue circle) has a shallow ledge with the substrate shape to embed it. The ledge is easier to visualize in the perspective view of the holding arm shown in panel B. The image in panel C shows a magnified view of the substrate holder. The 4 holes next to the holder allow fixing the substrate through two aluminum sheets (represented by dashed lines) attached to the holder by M3 shortened screws. The M3 screws must be shortened to a length not longer than 4 mm to avoid any contact with the X/Y/θ stage when lifting the Z-stage. The design of the holding arm can be adapted to substrates of different shapes and sizes. (For interpretation of the references to colour in this figure legend, the reader is referred to the web version of this article.)

The substrate holder is one of the main features of the aligner, allowing the placement of the rigid substrate in the upper position (Fig. 2). Thus, the microscope focus can be kept on the rigid substrate surface during the entire alignment process. This is an advantage when the rigid substrate has smaller microfabricated structures than the PDMS layer, and these structures can be used as a reference for guiding the alignment. Although the holder was designed for a 20 mm square glass substrate, it is easily interchangeable or adapted for substrates of different sizes and shapes.

In this design, it is required that the substrate on the holding arm is transparent. Although this might be a limitation, it can be easily overcome since usually at least one of the substrates to be aligned is transparent. For instance, if an opaque substrate needs to be aligned with a PDMS microfluidic chip, the opaque substrate can be placed on the X/Y/θ-stage while the PDMS chip is located on the holding arm. For this purpose, the holding arm can be modified to hold a variety of substrates, shapes and sizes without needing to make further changes in the instrument.

Our aligner can be decomposed in three main parts:

*The main support*. It consists of the base and the arm support. Both pieces support the entire upper portion of the aligner as well as the stages (Fig. 1). The Z-stage is directly screwed on the base (Table 1). On top of the Z-stage a stage adapter is attached which contains a LED lamp for illumination. The X/Y/θ stage is screwed on top of the stage adapter and is where the PDMS layer is placed during the alignment process.

**Table 1:** List of parts to be fabricated with a milling machine or 3D printer (dataset can be found in http://dx.doi.org/10.17632/jpxw5dph27.1).

Design file name	File type	Open source license	Location of the file	Estimated cost
*Base (item 7 in*Fig. 3*.)*	Plans	*GNU General Public License (GPL)*	http://dx.doi.org/10.17632/jpxw5dph27.1	*$ 150*
*Stage adapter (item 4 in*Fig. 3*)*	Plans	*GNU General Public License (GPL)*	http://dx.doi.org/10.17632/jpxw5dph27.1	*$ 150*
Holding arm (item 11 in Fig. 3)	Plans	*GNU General Public License (GPL)*	http://dx.doi.org/10.17632/jpxw5dph27.1	*$ 135*
Alternative holding arm designs	Plans	*GNU General Public License (GPL)*	http://dx.doi.org/10.17632/jpxw5dph27.1	*$ 135*
Arm support (item 6 in Fig. 3)	Plans	*GNU General Public License (GPL)*	http://dx.doi.org/10.17632/jpxw5dph27.1	*$ 145*
Aluminum sheets (item 12 in Fig. 3)	CAD	*GNU General Public License (GPL)*	http://dx.doi.org/10.17632/jpxw5dph27.1	*$ 1*
Raspberry Pi case.	CAD	*Creative Commons - Atribution*	http://dx.doi.org/10.17632/jpxw5dph27.1	*$ 0.5*
*The holding arm*. This piece is screwed on top of the arm support (Fig. 1) and holds the substrate in place using two aluminum sheets (Fig. 2).
*The digital microscope*. The microscope is located on top of the holding arm and it is connected to a Raspberry Pi board. Microscope visualization can be performed using a tablet, smartphone or computer. This means that the alignment process can be monitored remotely through any device with internet connection.

The main features of our aligner can be described as follow:

• The aligner allows the assembly of microfluidic devices by aligning two layers. These layers can be made of different materials. During the alignment, the substrate in the holder is kept still, whereas the material on the X/Y/θ-stage is moved to achieve correct positioning of both layers.
• The compact size of the aligner makes it portable and suited for small spaces. It can be stored in a drawer and carried without disassembling it.
• Microscopic visualization of the alignment process can be achieved with a smartphone connected to the digital microscope through a Raspberry Pi board.
• The substrate holder is easily interchangeable for the assembly of substrates of different size and shapes.
• The X/Y/θ-stage and the microscopic resolution provide the necessary accuracy and precision for the fabrication of microfluidic devices.

## Design files summary

**Fig. 3:**
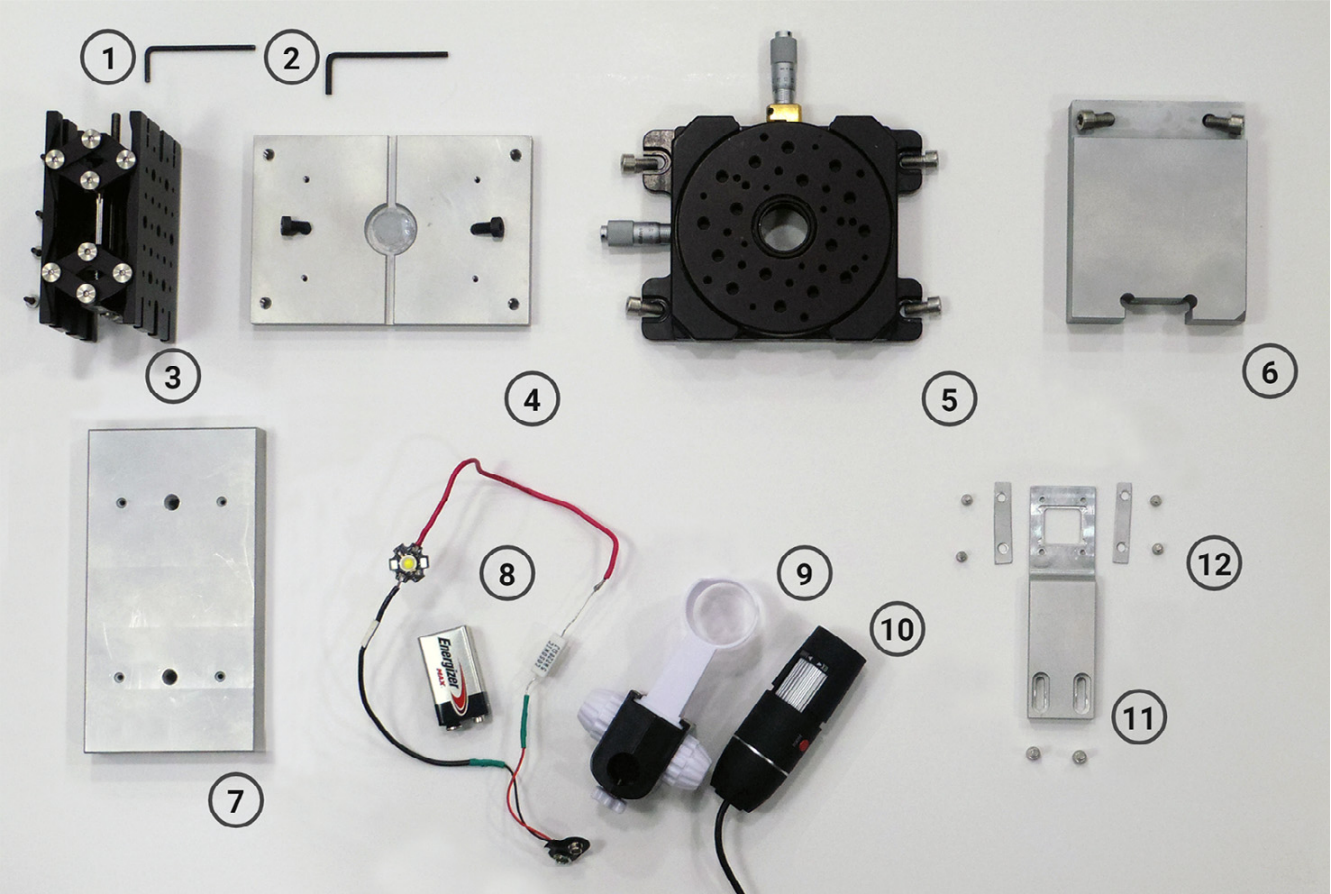
Parts for the assembly of the aligner (Raspberry Pi board and its case are not shown). 1) M3 Hex Allen key (for adjusting the aluminum sheets in the substrate holder). 2) 1/8″ Hex Aleen key (for moving the Z-stage). 3) Z-stage. 4) Stage adapter with two M6 screws. 5) X/Y/θ-stage with four M6 screws. 6) Arm support with two M6 screws. 7) Base. 8) Illumination circuit. The 5 W LED lamp is connected in series with a 220 Ω resistor. The circuit is fed by a 9 V battery. 9) Microscope holder. 10) Digital microscope. 11) Holding arm with two M4 screws. 12) Aluminum sheets and four shortened M3 screws.

The different parts that constitute the aligner (with the exception of the Raspberry Pi board) are shown in Fig. 3. The base of the aligner, the stage adapter, the holding arm and the arm support (items 7, 4, 11 and 6, respectively) were fabricated in 6061-T6 aluminum alloy using a computer numerical control (CNC) milling machine LAGUN GVC 600, equipped with a Fagor 8055 controller. The milling process was programmed by a computer aided manufacturing (CAM) software (BobCAD-CAM v32), which generated the G-Code for the Fagor controller. After machining, the surfaces were bead-blasted using 50–100 µm glass beads. Z-stage and X/Y/θ-stage were purchased from Thorlabs (Newton, NJ, USA) (items 3 and 5, respectively). Illumination was made by a simple circuit where a 5 W white LED lamp was connected in series with a 220 Ω resistor and a 9 V battery (item 8). The cost for the machined pieces was estimated at https://www.hubs.com/, although in our case, they were manufactured in the workshop of our university.

The base, stage adapter, arm support and holding arm in Table 1 were machined from a 500 mm long, 100 mm width and 25 mm thick aluminum plate. The aluminum plate had a cost of $ 80. The base supports all the components of the aligner. The stage adapter connects the Z-stage with the X/Y/θ-stage and houses the illumination lamp. The arm support is attached to the base and supports the holding arm. The aluminum sheets located in the substrate holder are used to keep the substrate in position during the aligning process. As mentioned above, the holding arm can be modified to hold substrates of different shapes and sizes. In the file “Alternative holding arm designs”, users can find holders for circular and rectangular glass cover slides. These holders can be employed for bonding PDMS to different glass substrates and represent an example of how the holding arm piece can be customized for different applications (Fig. S2).

## Bill of materials summary

**Table:** 

**Designator**	**Component**	**Number**	**Cost per unit -currency**	**Total cost -currency**	**Source of materials**
*Z-stage (item 3 in*Fig. 3*)*	*Jack for controlling the Z position during the aligning. It includes a Hex Allen key for moving.*	*1*	*$ 473.72*	*$ 473.72*	Product link
X/Y/θ-stage (item 5 in Fig. 3)	*XY stage with rotational control*	1	*$ 695.45*	*$ 695.45*	Product link
Lamp	*5 W LED white lamp*	1	*$ 1*	*$ 1*	Product link
Resistor	COSONIC 220 Ω ceramic resistor	1	*$ 1*	*$ 1*	Product link
Allen Key set	M3, M4 and M6 Allen keys	1	*$ 10*	*$ 10*	Local hardware store
Shortened M3 screw (4 mm long)	M3 socket head cap screw	4	*$ 0.1*	*$ 0.4*	Local hardware store
M3 screw	M3 socket head cap screw	4	*$ 0.1*	*$ 0.4*	Local hardware store
M4 screw	M4 socket head cap screw	2	*$ 0.1*	*$ 0.2*	Local hardware store
M6 screw	M6 socket head cap screw	8	*$ 0.1*	*$ 0.8*	Local hardware store
Digital microscope (item 10 in Fig. 3)	Digital microscope GADNIC MICROS07	1	*$ 50*	*$ 50*	Product link
Microscope holder (item 9 in Fig. 3)	Holder for the microscope GADNIC MICROS07. (Included with the microscope)	1			
Single-board computer	Raspberry Pi board	1	*$ 250*	*$ 250*	Product link
Battery	9 V battery	1	*$ 1*	*$ 1*	Local hardware store
Battery connector	9 V battery connector	1	*$ 0.5*	*$ 0.5*	Product link

The estimated total cost of the aligner including the aluminum machined parts is $ 2065.

## Build instructions

**Fig. 4:**
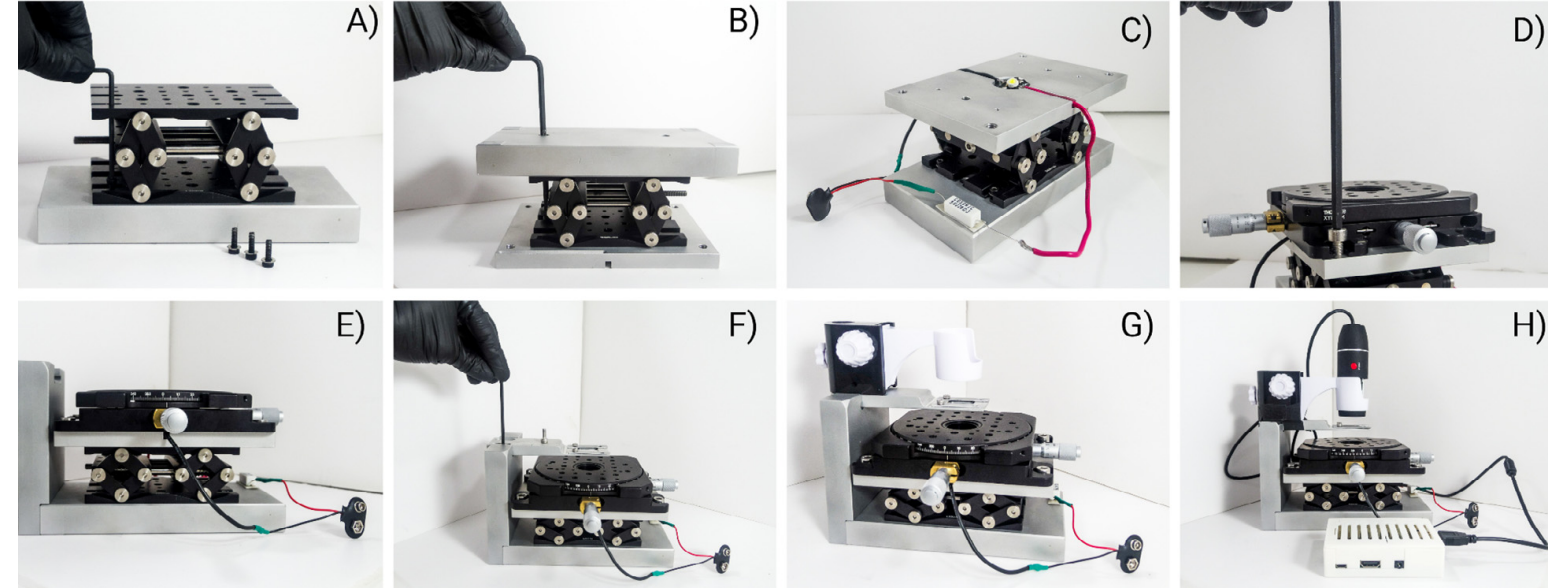
Building instructions for the aligner. First, attach the Z-stage to the base with M3 screws (panel A). Then, place the stage adapter with the lamp cavity down. Turn upside down the Z-stage and the base and place them on the stage adapter. Attach the stage adapter to the Z-stage using M6 screws (panel B, note that the base has two holes for the M6 key). Return the aligner to the upright position and add the lamp circuit. Glue the lamp with thermally conductive silicon glue in the cavity of the stage adapter (panel C). Screw the X/Y/θ-stage on top of the stage adapter with four M6 screws (panel D). Attach the arm support to the back side of the base with two M6 screws (panel E). Screw the holding arm on the arm support with two M4 screws (panel F). Place the microscope holder on the holding arm (panel G). Connect the Raspberry Pi board (panel H).

4.1 Attach the Z-stage to the base using four M3 socket head cap screws (Fig. 4A). Place the 1/8″ hexagonal socket for adjusting the height of the Z-stage facing the front of the aligner.

4.2 Place the stage adapter with the lamp cavity facing down. Place the Z-stage attached to the base upside down on top of the stage adapter as shown in Fig. 4B. Screw the stage adapter to the Z-stage using two M6 screws. Note that the base has two holes to allow the Allen key to reach the stage adapter from above.

4.3 Return the device to the upright position. The lamp cavity in the stage adapter should be facing up. Prepare the illumination lamp circuit by soldering the lamp and resistor in series with a 9 V battery connector. Place the lamp in the cavity in the stage adapter and glue it to the aluminum surface using thermally conductive silicone glue for mounting LEDs. The wires can be placed inside the channels located at both sides of the lamp cavity (see Fig. 4C). The resistor of the circuit can be fixed on the base using double-sided tape.

4.4 Screw the X/Y/θ-stage on top of the stage adapter using four M6 screws as shown in Fig. 4D. Place the X/Y/θ-stage with both micrometer adjusting screws facing the front and the left side of the aligner.

4.5 Lower the Z-stage to its lowest position. Screw the arm support to the base using two M6 screws (Fig. 4E).

4.6 Screw the holding arm on the arm support with two M4 screws as shown in Fig. 4F.

4.7 Attach the microscope holder to the holding arm (Fig. 4G) using silicone glue or tape. To find the correct position for the microscope holder place a substrate in the substrate holder and find the position of the microscope that allows a correct visualization of the substrate features. Removing the microscope plastic cap allows to set the microscope closer to the substrate.

4.8 Place the Raspberry Pi on the back of the arm support using double-sided tape. Connect the microscope to the USB port of the Raspberry Pi board (Fig. 4H).

## Operation instructions

**Fig. 5:**
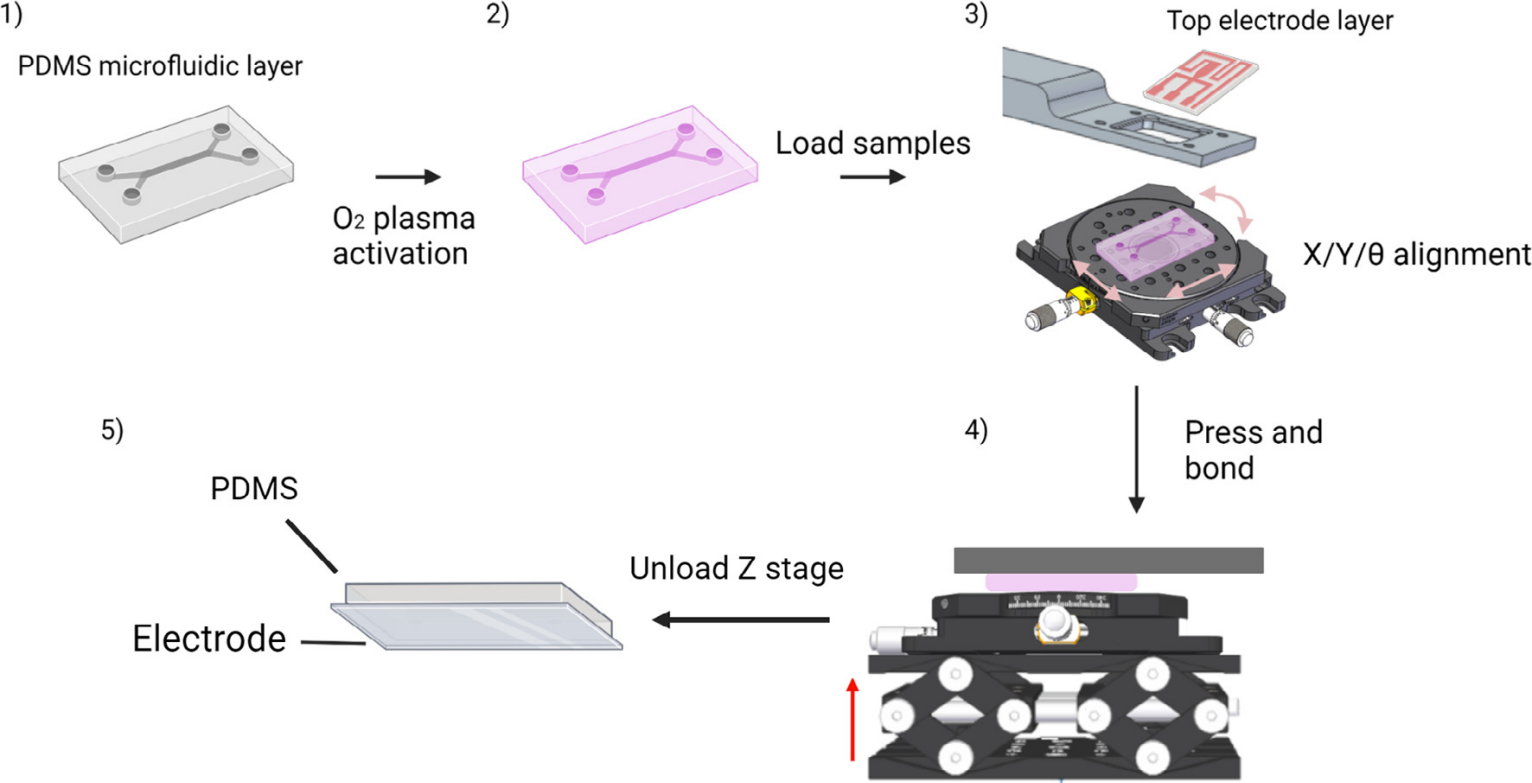
A simplified scheme of the alignment procedure. The PDMS and ITO substrate are activated with O_2_ plasma treatment (panels 1 and 2). Then, the ITO substrate and PDMS are placed in the holder and on the X/Y/θ-stage, respectively. The PDMS chip is aligned with the ITO substrate by moving the substrate in the X, Y and θ dimensions (panel 3). Once alignment is achieved, the Z-stage is lifted to achieve full contact between the substrates (panel 4). After 5 min, the Z-stage is lowered, and the assembled chip is removed (panel 5).

Fig. 5 shows a scheme for the alignment of a PDMS microfluidic chip with an ITO microelectrode on a glass substrate (ITO substrate). A similar procedure can be followed for aligning other types of substrates. First, both PDMS microfluidic chip and ITO substrate are subjected to O_2_ plasma treatment. The ITO substrate is placed on its corresponding holder, whereas the PDMS chip is placed on a glass slide on top of the X/Y/θ-stage (Fig. 5). Note that the substrates to be bonded must be facing each other during the alignment. The substrates are aligned using the X/Y/θ-stage, then the Z-stage is lifted to achieve full contact between the activated surfaces. Both substrates are kept in contact for 5 min, then the Z-stage is lowered, and the bonded substrates are retrieved.

**Fig. 6:**
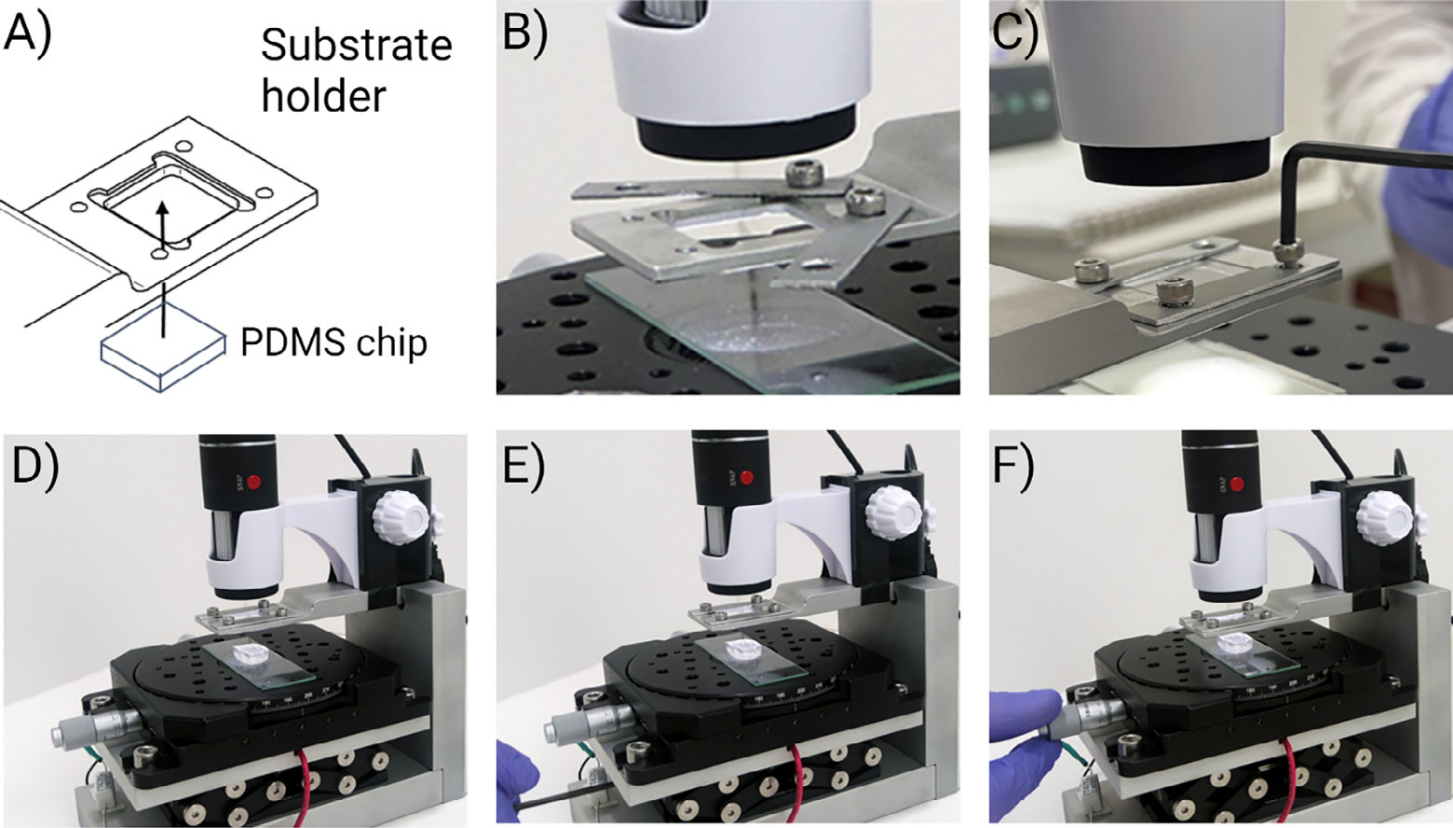
Alignment procedure using the aligner instrument. The PDMS must be small enough to pass through the opening in the holder to contact the ITO substrate (panel A). Before alignment, prepare the holder and glass slide to place the substrates as shown in the image in panel B. After O_2_ plasma activation place the ITO substrate in the holder and adjust the aluminum sheets with the shortened M3 screws (panel C). Place the PDMS substrate on the glass slide on the X/Y/θ-stage. Manually align the PDMS with the ITO substrate (panel D). Lift the Z-stage with the Allen key to bring closer together both substrates (panel E). Adjust the position of the PDMS with the micrometer adjusting screws and rotating disk of the X/Y/θ-stage (in panel F).

Before the alignment, it is crucial to cut the PDMS chip to a size smaller than the opening in the substrate holder to allow contact between both substrates (Fig. 6A). Ensure that the focus of the microscope is set in the proper plane of the ITO substrate by adjusting the magnification and position of the microscope holder. The resolution of the microscope was sufficient to distinguish features of 10 µm in size on both substrates. The digital microscope can be calibrated using a small grid as shown in Fig. S1.

In our experimental conditions, the alignment procedure has to be completed within 10 min after O_2_ plasma activation. At longer times, weak bonding between PDMS and ITO substrate are obtained which results in leakage and inadequate performance of the assembled device. We strongly recommend performing an alignment test prior O_2_ plasma activation to ensure that bonding between substrates can be performed within the recommended time frame.

The following steps describe the process in which an ITO substrate is bound to a PDMS microfluidic chip. The microscope monitoring of the process is shown in video S1.

1. Connect a smartphone or tablet to the microscope through the remote control of the Raspberry Pi. For this, follow the instructions in the Readme.txt file in the data repository (http://dx.doi.org/10.17632/jpxw5dph27.1)
2. Connect the 9 V battery to start the illumination. Prepare the substrate holder for the ITO substrate and the glass slide for the PDMS (Fig. 6B)
3. Perform O_2_ plasma activation on the ITO substrate and PDMS.
4. Place the ITO substrate in the substrate holder of the holding arm. The O_2_ plasma-activated surface must be facing down.
5. Fix the position of the ITO substrate using the aluminum sheets (Fig. 6C).
6. Assure that the microscope focus is set on the electrode surface.
7. Set the Z-stage to its lowest position (Fig. 6D).
8. Place the PDMS chip on a microscope glass slide in the center of the X/Y/θ-stage. The O_2_ plasma-activated PDMS surface must be facing up (Fig. 6D).
9. Lift the Z-stage until the distance between the PDMS and the ITO substrate is approximately 2 mm (Fig. 6E). Make sure that the position of the PDMS chip is such that it can pass through the opening in the substrate holder.
10. Adjust the position of the PDMS layers using the micrometer adjusting screws and the rotating disk of the X/Y/θ-stage (Fig. 6E). After completing this step, both layers should be aligned.
11. Lift the Z-stage while maintaining the alignment between layers until reaching contact. Use the X/Y/θ-stage to make the necessary adjustments during the whole process. Adjust the focus of the microscope when necessary. The process can be seen in video S1.
12. Once both layers are in contact, we recommend keeping them in that position for at least 5 min to ensure a strong bonding.
13. Lower the Z-stage to its lowest position. Release the aluminum sheets that hold the ITO substrate and retrieve the assembled microfluidic device.

Safety hazards: Caution is recommended when lifting or lowering the Z-stage. Avoid putting fingers or hair inside the stage that could get trapped. Be cautious when the PDMS chip and the ITO substrate are in contact. If the applied pressure is too high, the glass may break. To prevent this, frequently check the distance between layers. After contact is reached, a slight increase in pressure is sufficient to achieve bonding between layers. Wear eye protection.

If one of the substrates breaks, we recommend cleaning the device with care and removing the glass debris with adhesive tape before operating it again. Keep the surface of the X/Y/θ-stage clean, as tiny debris may make the surface uneven and tilt the PDMS substrate. This will hamper full contact between both layers.

## Validation and characterization

**Fig. 7:**
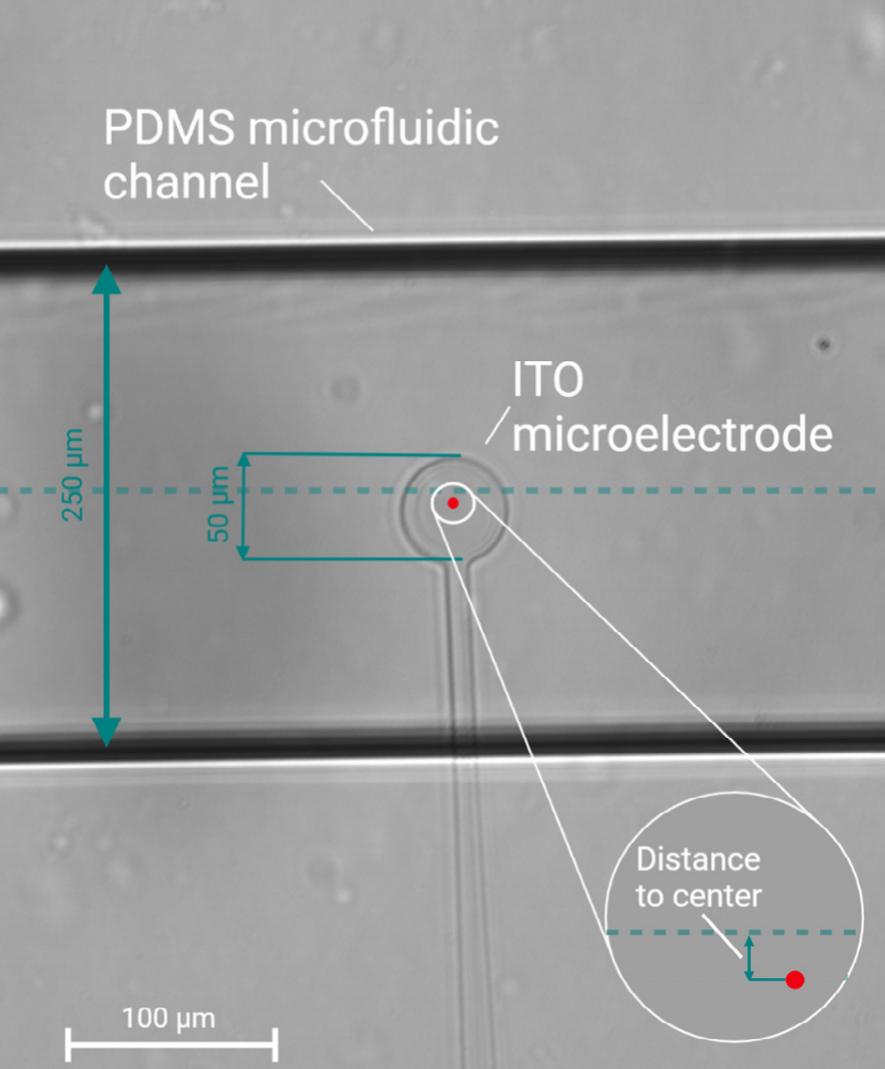
Micrograph of the assembled microfluidic device with incorporated electrodes. The image shows an ITO electrode of 50 µm diameter. The electrode was placed inside a 250-µm-width PDMS channel. The green dashed line represents the channel center. The red dot represents the center of the electrode. The image was obtained by bright field microscopy. (For interpretation of the references to colour in this figure legend, the reader is referred to the web version of this article.)

The performance of the aligner was tested by aligning microfluidic PDMS chips with 50- and 100-μm-diameter ITO microelectrodes fabricated on a glass substrate. The fabrication and characterization of the microelectrodes employed in this work is published elsewhere [18]. The objective was to locate the center of the circular microelectrode in the center of 250-μm-width microfluidic channel (Fig. 7). Images of the aligned devices were taken using a high-resolution microscope (Zeiss Led 1P). To determine the precision and accuracy of each alignment we measured the distance from the center of the electrode to the middle of the channel according to equation (1):

(1) $$\mathrm{Distance}=\left|ax+b-y\right|/\sqrt{\left(a^{2}+1\right)}$$

where *x* and *y* are the coordinates of the center of the electrode. *a* and *b* are the slope and the y-intercept, respectively, of the equation of the dashed line representing the middle of the channel (Fig. 7). The line representing the middle of the channel was estimated using ImageJ.

**Fig. 8:**
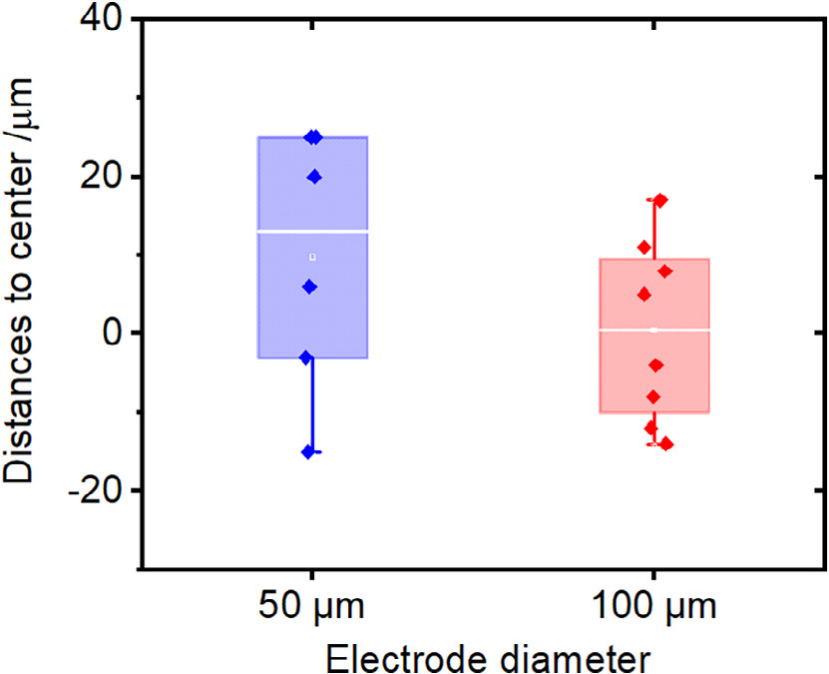
Results from the accuracy and precision test of the aligner. The points in the plot represent the measured distances between the ITO electrode center and the middle of the PDMS channel. The box size represents the range from the 25th to the 75th percentile of the sample population. The error bars represent the range from the 5th to the 95th percentile of the sample population. The white line inside the box represents the median of the population. N = 6 for 50 μm electrodes and N = 8 for 100 μm electrodes.

The average distance from the center of the electrode to the middle of the channel was 0.4 μm and 9.7 μm for the 100- and 50-μm-diameter ITO microelectrodes, respectively (Fig. 8), which is near the resolution of the digital microscope (Fig. S1). The standard deviation of the measurements was 11.5 μm and 16.5 μm for the electrodes of 100 μm and 50 μm, respectively (Fig. 8). The deviation of the 50-μm electrodes was slightly higher, probably due to its smaller size, which made it more difficult to estimate their relative position with respect of the center to the channel. This can be understood in the context of the Weber-Fechner law, which describes the relation between the actual change in a stimulus and how humans perceive that change [19]. The law states that the change in a stimulus that will be just noticeable is a constant ratio of the original stimulus. To put it in simple words, the larger the diameter of the electrode is, the proximity of its perimeter to the channel wall increases, which makes it easier to estimate its position inside the channel. Therefore, the difference in accuracy for the different alignment procedures might arise from the user’s perception, more so than hardware performance.

The alignment performance can be calculated from the data in Fig. 8. In the case of the 100 μm electrodes, half of the devices had a misalignment smaller than 9 μm, while no value above 17 μm was obtained for the total of the aligned devices. The misalignment errors are near the digital microscope resolution (Fig. S1), which suggest that the aligner accuracy is limited by the digital microscope capabilities. Moreover, we haven’t observed differences in alignment that can be attributed to variations in PDMS thickness, ranging from 1 to 6 mm. Nevertheless, the accuracy of our aligner is suited for microfluidic applications and it is similar to that of the design of Li et al. [14] and He et al. [17]. In contrast, the aligner proposed by Kipper et al. [15] showed a higher accuracy as the misalignment error of their aligner was 4 ± 3 µm. The authors achieved this accuracy by incorporating a sophisticated semi-automated method of alignment in their device. In our case, we aimed to create a cost-effective, and easy to replicate design, therefore complex or expensive complements were omitted. A key aspect for improving the accuracy of our aligner would be to use a microscope with higher resolution as mentioned before. Users may evaluate whether it would be worth investing in a microscope with better capabilities based on their applications.

The assembled microfluidic devices were evaluated by means of electrochemistry. Cyclic voltammetry was performed at a scan rate of 10 mV/s in PBS buffer containing K_3_[Fe(CN)_6_] 1 mM as redox probe at a flow rate of 5 μl/min (Fig. S3). A typical microelectrode response was observed where the steady-state current is proportional with the diameter of the electrode [20], indicating that the fabrication and alignment processes were satisfactory.

Finally, we tested whether our aligner can be employed for creating other types of microfluidic devices. Fig. S4 shows micrographs of different composite microfluidic chips combining ITO microelectrodes on glass with PDMS that were created using the aligner. Fig. S4 also shows how the aligner can be employed to align two PDMS layers together after O_2_ plasma treatment. Video S2 shows the monitoring of this alignment process using the digital microscope. Fig. S5 shows that visualization of the aligning process can be done even if one of the substrates is opaque. Taken together, these results highlight the versatility and robustness of the aligner for the fabrication of a variety of microfluidic devices involving different materials.

The fabrication of microfluidic devices with incorporated microelectrodes would not have been possible without the aligner presented in this work. This aligner has become a key tool for the fabrication of microfluidic devices in our laboratory. We hope that our design can be of use for many research groups and education establishments interested in microfluidics and microfabrication.

## Declaration of Competing Interest

The authors declare that they have no known competing financial interests or personal relationships that could have appeared to influence the work reported in this paper.
