# Supplementary material for: A portable and affordable aligner for the assembly of microfluidic devices
Source: HardwareX. 2022 Aug 27;12:e00348. doi: 10.1016/j.ohx.2022.e00348 (PMC9465365; doi:10.1016/j.ohx.2022.e00348)
Supplement: Supplementary data 1 [file mmc1.docx]

**Appendix 1**


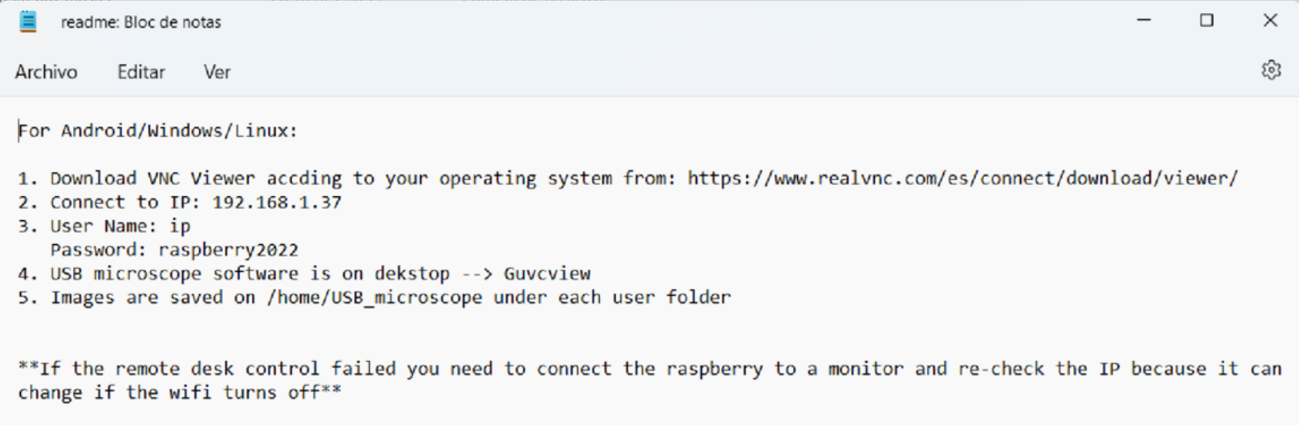


**Appendix 1**. Instructions for remote access to the Raspberry Pi that controls the USB-digital microscope. The software allows the user to take photos and films during the alignment procedure. This file is available at [*https://data.mendeley.com/datasets/jpxw5dph27/draft?a=49cd70f9-b629-4801-85f0-3dcce4f0344f*](https://data.mendeley.com/datasets/jpxw5dph27/draft?a=49cd70f9-b629-4801-85f0-3dcce4f0344f)*.*

**Supplementary Table 1**

A comparison between different aligners with similar designs

| Article | Cost | Tested on | Alignment error | More relevant features |
| --- | --- | --- | --- | --- |
| [Li et al. (2015)](https://aip.scitation.org/doi/full/10.1063/1.4927197) | Not declared | PDMS multilayered devices | - Translational accuracy between 8.2-13.6 µm - Rotational error between 0.11-0.14° | - Two digital microscopes to have a wide field of view - A glass slide is used to hold the PDMS in the upper position |
| [He et al. (2021)](https://www.sciencedirect.com/science/article/pii/S2452199X2100582X) | Not declared | PDMS multilayered devices | - Alignment accuracy: 10 μm | - Stereo microscope for visualization - A glass slide is used to hold the PDMS in the upper position |
| [Kipper et al. (2017)](https://pubs.rsc.org/en/content/articlelanding/2017/lc/c6lc01534d/unauth) | Not declared | PDMS-glass and PDMS multilayered devices | - Translational accuracy: 4 µm - Rotational error: 0.003° | - Semiautomatic computer aided alignment - Two types of holders for the PDMS |
| [Kim et al. (2005)](https://www.sciencedirect.com/science/article/pii/S0924424704007071?casa_token=yt3TFeSrGsEAAAAA:0RMaYVWVY2OgV0de7Evor_IoLlg5QX0f-0Qqi836rexj7R9-fbfeMSqHd4XcwMCuLuWTU6tzqW0) | Not declared | PDMS multilayered devices | - Alignment accuracy: 2 μm | - Vacuum holder for the substrate in the lowest position and a pin holder for the substrate in the upper position - Stereo microscope for visualization |
| This work | $ 2065 | PDMS-ITO electrodes on glass substrates | - Alignment accuracy: 11.5 μm (limited by the digital microscope resolution) | - A holder in the upper position for the ITO electrode - A digital microscope for visualization - Microscopy visualization is performed using a Raspberry Pi connected to a mobile device |

**Supplementary Figure 1**


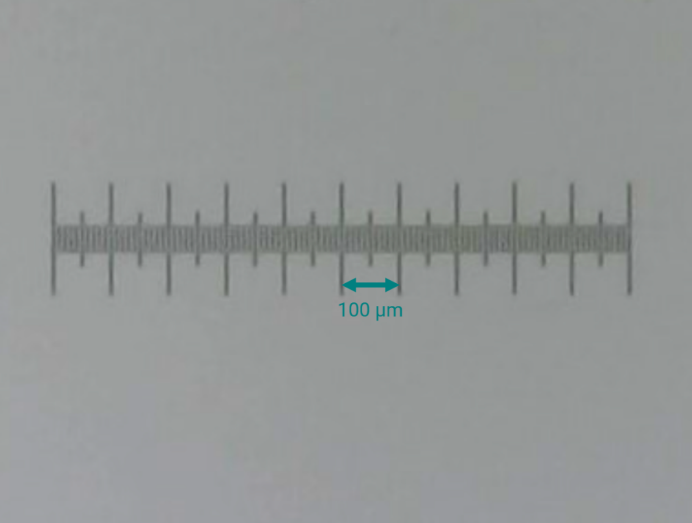


***Fig. S1.*** *The image shows a microscale for the calibration of the digital microscope employed in the aligner device. The microscale was placed in the substrate holder and the image was taken by the digital microscope. The long bars of the scale are separated by 100 μm as shown in the image. The short bars are separated by 50 μm. Between the bars there are marks inside the scale every 10 μm.*

**Supplementary Figure 2**


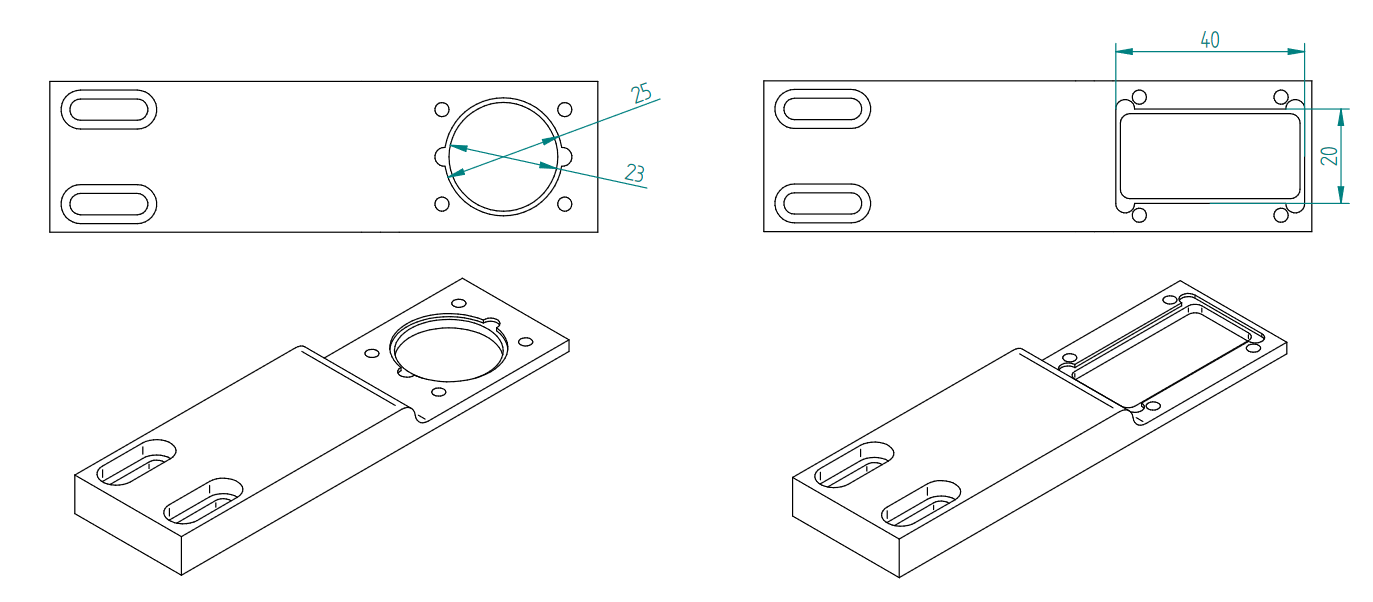


A)

B)

***Fig. S2.*** *Panel A shows a holding arm adapted to hold a 25 mm-diameter glass cover slide. Panel B is a design for a 40x20 mm glass slides. These holder arms can be used with the same aligner to align different devices using different substrates.*

**Supplementary Figure 3**

**
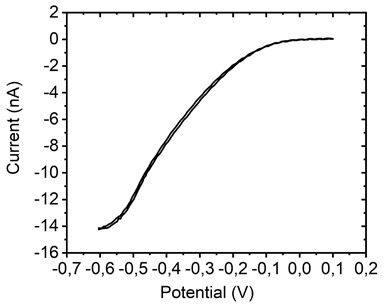
**

***Fig. S3.*** *Cyclic voltammogram (ν_scan_ = 10 mV/s) in PBS buffer containing 1 mM K3[Fe(CN)6] for a 100-µm ITO microelectrode inside the microfluidic channel at a flow rate of 5 μl/min.*

**Supplementary Figure 4**

**
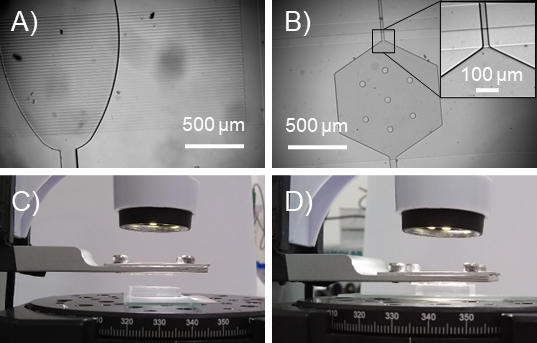
**

***Fig. S4.*** *Panel A shows an image of a microfluidic device comprising an array of 10 μm-wide interdigitated ITO electrodes separated by 20 μm and an ellipsoidal microfluidic chamber. Panel B shows an image of an ITO microelectrode that was aligned with a hexagonal PDMS microfluidic chamber. The inset shows the precise alignment between the microelectrode border and the vertex of the angle between the hexagon’s side and the lateral channel of the PDMS chamber. Panels C and D show the procedure for the alignment of two PDMS substrates. After O_2_ plasma treatment, one of the PDMS substrates is reversibly bound to a 20x20 mm glass slide by contacting the face not activated by O_2_ plasma. The glass slide with the bonded PDMS is then placed in the substrate holder with the PDMS chip facing down. For this particular substrate holder, the PDMS substrate must be 18x18 mm or smaller with a thickness of at least 2 mm. The second PDMS chip is placed on a glass slide on the X/Y/θ-stage with the O_2_-plasma-activated side facing up as shown in panel C. Finally, substrates are aligned as described in section 5. Panel D shows an image of the aligned PDMS substrates. A video of the aligning process monitored using the digital microscope is presented in Video S2.*

**Supplementary Figure 5**


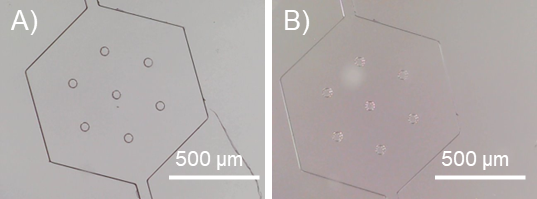


***Fig. S5.*** *Panel A shows an image of a PDMS substrate in the holding arm acquired with the digital microscope. The working distance of the digital microscope is approximately 30 mm, which make it easy to focus on 5 mm-thick PDMS substrates. In case the substrate on the X/Y/θ-stage is opaque, illumination can be achieved using the digital microscope LED lamps. Panel B shows the image of the same PDMS substrate shown in panel F but bonded to an opaque substrate placed on the X/Y/θ stage.*
